# The Design of a Low-Power Pipelined ADC for IoT Applications

**DOI:** 10.3390/s25051343

**Published:** 2025-02-22

**Authors:** Junkai Zhang, Tao Sun, Zunkai Huang, Wei Tao, Ning Wang, Li Tian, Yongxin Zhu, Hui Wang

**Affiliations:** 1 Shanghai Advanced Research Institute, Chinese Academy of Sciences, Shanghai 201210, China; zhangjk@sari.ac.cn (J.Z.);; 2 University of Chinese Academy of Sciences, Beijing 100049, China; 3 Synopsys Semiconductor Technology (Shanghai) Co., Ltd., Shanghai 200433, China

**Keywords:** pipelined analog-to-digital converter (ADC), low power, sample-and-hold amplifier-less (SHA-less), op-amp sharing

## Abstract

This paper proposes a low-power 10-bit 20 MS/s pipelined analog-to-digital converter (ADC) designed for the burgeoning needs of low-data-rate communication systems, particularly within the Internet of Things (IoT) domain. To reduce power usage, multiple power-saving techniques are combined, such as sample-and-hold amplifier-less (SHA-less) architecture, capacitor scaling, and dynamic comparators. In addition, this paper presents a novel operational amplifier (op-amp) with gain boosting, featuring a dual-input differential pair that enables internal pipeline stage switching, effectively alleviating the crosstalk and memory effects inherent in conventional shared op-amp configurations, thereby further reducing power consumption. A prototype ADC was fabricated in a 180 nm CMOS process and the core size was 0.333 mm^2^. The ADC implemented operated at a 20 MHz sampling rate under a 1.8 V supply voltage. It achieved a spurious-free dynamic range (SFDR) of 61.83 dB and a signal-to-noise-and-distortion ratio (SNDR) of 54.15 dB while demonstrating a maximum differential non-linearity (DNL) of 0.36 least significant bit (LSB) and a maximum integral non-linearity (INL) of 0.67 LSB. Notably, the ADC consumed less than 5 mW of power at the mentioned sampling frequency, showcasing excellent power efficiency.

## 1. Introduction

**Figure 1:**
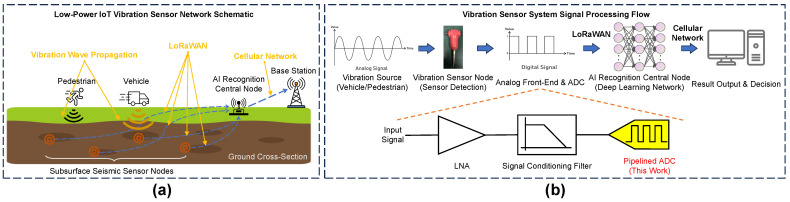
An overview of a low-power IoT vibration sensor network. (**a**) Subsurface seismic sensor deployment for vehicle/pedestrian vibration detection in a smart city, with LoRaWAN communication to an AI node. (**b**) Signal processing flow in a sensor node, highlighting the analog front-end and proposed low-power pipelined ADC for energy-efficient data conversion.

The rapid advancement of wireless communication technologies has led to the explosive growth of Internet of Things (IoT) devices, unlocking vast potential across a wide range of sectors [1,2]. These interconnected devices, typically equipped with various sensors and utilizing wireless communication protocols such as Bluetooth Low Energy [3], Wi-Fi [4], and LoRaWAN [5], are transforming data collection, transmission, and device-to-device communication, enabling seamless interaction with the physical world [6,7]. The rapid proliferation of IoT devices, which are projected to reach billions globally, is driving transformative advancements in areas such as smart homes, smart cities, industrial automation, and healthcare. This surge is not only enhancing efficiency and sustainability but also improving quality of life [8,9,10]. Figure 1a illustrates a typical IoT-based vibration monitoring system, showcasing a network of subsurface seismic sensor nodes deployed for vehicle and pedestrian vibration detection within a smart city environment.

A defining characteristic of many IoT deployments, particularly in sensor-rich applications such as environmental monitoring and infrastructure health management, is the stringent demand for ultra-low power consumption [11,12]. IoT devices are often deployed in remote and harsh environments, where they rely solely on limited battery power to maintain long operational lifespans, typically ranging from 2 to 3 years [13]. As such, energy-efficient design is not merely an optimization goal but a critical requirement for the widespread and sustainable deployment of IoT systems [14]. While optimizing low-power communication protocols is essential for reducing energy consumption, achieving truly ultra-low power operation necessitates a comprehensive approach involving low-power design and optimization at the hardware level [15,16]. Energy-efficient analog-to-digital conversion at the hardware level is crucial, as exemplified by the signal processing chain commencing with vibration sensing in a typical IoT sensor node, as shown in Figure 1b.

At the radio hardware level, power consumption is distributed across several key modules, including radio frequency transceivers, baseband processors, analog-to-digital converters (ADCs), and low-noise amplifiers (LNAs). Among these critical components, the ADC—serving as a crucial interface for digitizing analog sensor signals—has been consistently identified as a major contributor to power consumption in the signal processing chain, particularly in sensor-rich IoT nodes that require continuous data acquisition [17,18]. There are various ADC architectures, each characterized by trade-offs in terms of speed, resolution, and power efficiency. Successive approximation register (SAR) ADCs are commonly used for low-to-medium-speed applications, owing to their inherent power efficiency and moderate conversion speed [19]. Recent advancements, such as a 16-bit, 1 MS/s design by Ding et al. [20], have achieved an impressive 108.9 dB spurious-free dynamic range (SFDR) at 1 kHz through floating capacitor self-calibration and round-robin LSB grouping in 180 nm CMOS technology, all while consuming only 6.745 mW. In contrast, flash ADCs excel in achieving extremely high conversion speeds; however, this advantage comes at the expense of increased power dissipation and reduced resolution, making them less suited for energy-constrained IoT devices [21]. Recent designs, such as an eight-bit, 1-GSPS flash ADC with latency-optimized encoding [22], demonstrate improved linearity (7.01 effective number of bits, ENOB), but they still face inherent latency and area trade-offs. On the other hand, pipelined ADCs offer an optimal balance between speed and accuracy by dividing the conversion process into multiple stages, each handling a portion of the signal. This multi-stage architecture facilitates high throughput and precision, making pipelined ADCs particularly suitable for medium-to-high-speed applications in wireless communication and image processing-oriented IoT systems. Given these advantages, we selected the pipelined ADC for the analog front-end system in our energy-efficient data conversion approach. As shown in Figure 1b, our design outlines the signal processing flow within a sensor node, emphasizing the pivotal role of the pipelined ADC in optimizing performance while minimizing power consumption.

Recent advancements in pipelined ADCs have focused on improving resolution, speed, and power efficiency, often leveraging advanced process nodes. For example, Zhou et al. [23] demonstrated a 16-bit, 50 MS/s ADC in a 180 nm CMOS, achieving 70.2 mW with an SFDR exceeding 100 dB, highlighting the potential benefits of moderate process scaling. At higher speeds, Xu et al. [24] developed a 12-bit, 1 GS/s pipelined ADC in a 40 nm CMOS, optimizing settling time to reduce power consumption to 97.6 mW. In further high-speed designs, Zhang et al. [25] introduced a 12-bit, 1.25 GS/s RF-sampling ADC in a 28 nm CMOS, consuming only 45.6 mW. In contrast, Lagos et al. [26] achieved 81 dB SFDR at 1 GS/s using 16 nm FinFET technology, with a significantly lower power consumption of 17.8 mW, demonstrating substantial energy efficiency improvements. On the extreme end, Yang et al. [27] presented a 14-bit, 4 GS/s ADC in a 28 nm CMOS, and its power consumption reached 782 mW, highlighting the challenges of power management at ultra-high speeds. In contrast, Gao et al. [28] proposed a 12-bit, 600 MS/s ADC in a 28 nm CMOS, achieving an optimal balance between speed and power consumption at 15.6 mW, employing a two-stage gain-boosting FIA-based residue amplifier. However, for many IoT applications, particularly those involving low-data-rate sensing such as vibration monitoring, the emphasis on ultra-low power consumption often outweighs the need for gigahertz sampling rates or extremely high resolutions.

This work focuses on the design of a pipelined ADC for vibration detection in IoT systems, as illustrated in Figure 1. Vibration sensing, commonly employed in smart city infrastructure monitoring and industrial predictive maintenance, typically involves the acquisition of low-frequency signals. For these applications, a 10-bit resolution and a 20 MS/s sampling rate are sufficient to provide the necessary bandwidth and dynamic range for accurately digitizing the target vibration signals. In the context of distributed IoT sensor networks, energy efficiency and cost-effectiveness are crucial for ensuring long-term, maintenance-free operation and large-scale deployment. Utilizing a mature 180 nm CMOS process strikes a strategic balance between performance, power consumption, and cost, making it an optimal choice for power-constrained IoT sensor nodes. In comparison to more advanced yet costly and potentially higher-leakage processes, 180 nm CMOS offer a cost-effective platform for ultra-low-power designs.

In this paper, we design and implement a novel low-power 10-bit 20 MS/s pipelined ADC for IoT applications. The main contributions of this work can be summarized as follows:

(1) We propose a novel gain-boosted operational amplifier with a dual-input differential pair that is meticulously optimized for internal pipeline stage switching. This innovative operational amplifier (op-amp) architecture effectively mitigates switching crosstalk and memory effects, which are especially significant in the absence of a front-end sample-and-hold amplifier-less (SHA-less) architecture and in op-amp sharing configurations. As a result, the proposed design enhances signal integrity, improves linearity, and minimizes power consumption.

(2) In addition to the op-amp innovation, we present a highly optimized pipelined architecture that incorporates precision refinement techniques in each stage, along with a step-wise scaling of sampling capacitor sizes. By eliminating the power-intensive front-end sample-and-hold amplifier (SHA) and implementing a shared op-amp architecture across multiple stages, our design significantly reduces overall power consumption.

(3) The proposed ADC architecture and circuit techniques were validated through a prototype fabricated in standard 180 nm CMOS technology. Measurement results demonstrate that the ADC achieved excellent performance metrics while consuming less than 5 mW at a 20 MS/s sampling rate. Furthermore, the prototype occupied a chip area of only 0.333 mm^2^, significantly smaller than typical designs implemented in more advanced process nodes.

This paper is organized as follows: Section 2 provides a detailed description of the architecture of the proposed low-power pipelined ADC. Section 3 discusses the key low-power design techniques employed in this work. Section 4 offers an in-depth examination of the specific circuit design implementation and critical design considerations. Section 5 presents experimental results and performance analysis, including a comprehensive comparison with state-of-the-art ADCs. Finally, Section 6 concludes the paper and outlines potential directions for future research.

## 2. Architecture of Pipelined ADC

**Figure 2:**
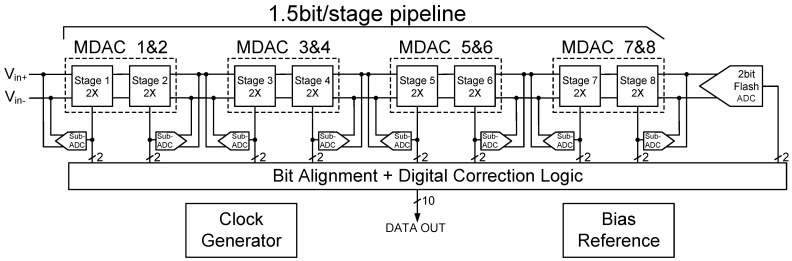
Architecture of 10-bit pipelined ADC.

The overall architecture of the proposed 10-bit pipelined ADC is depicted in Figure 2. The core structure of the 10-bit ADC comprises an eight-stage pipeline with 1.5 bits per stage, followed by a final 2-bit flash ADC. Each pipeline stage includes a multiplying digital-to-analog converter (MDAC) and a sub-analog-to-digital converter (sub-ADC). To optimize area and power efficiency, the first eight pipeline stages share amplifiers between adjacent stages, requiring only four op-amps to implement the entire 10-bit pipelined ADC. This design significantly reduces both the chip area and power consumption. Furthermore, a key feature of this architecture is the elimination of the traditional SHA structure, further contributing to power reduction. The final stage uses a flash ADC to directly quantize the last two bits of the input signal without the need for additional amplification stages, thus enhancing the overall conversion speed. The digital correction module, consisting of a register array and an adder, performs digital correction and encoding on the outputs of each stage to compensate for gain mismatches and offset errors between stages. This correction improves the overall linearity of the ADC. Additionally, the prototype circuit includes an on-chip clock generator to produce two-phase overlapping clocks, as well as an on-chip reference circuit to generate bias voltages for each stage. Detailed circuit implementation will be presented in an upcoming paper.

The operation of the 10-bit pipelined ADC proceeds as follows: The analog input signal first enters the initial pipeline stage, where the sub-ADC performs preliminary quantization, outputting a two-bit digital signal. The least significant bit (LSB) serves as a redundant bit for digital error correction to mitigate the effects of comparator offset voltage. The sub-digital-to-analog converter (sub-DAC) within the MDAC then converts this two-bit digital signal into an analog signal, generating a residue signal corresponding to the input signal. This residue is amplified by the multiplying gain unit within the MDAC and passed to the next pipeline stage for further processing. This process is repeated across the first eight 1.5-bit stages, with each stage contributing to the overall conversion precision. In the final stage, a two-bit flash ADC performs the rapid quantization of the residue signal. The digital output codes are then delayed and aligned before being processed by the digital correction circuit, which produces the final 10-bit digital output, completing the analog-to-digital conversion of the input signal.

## 3. Low-Power Techniques

This section highlights the various low-power techniques integrated into the architecture of the proposed ADC. The strategic combination of these approaches results in a highly power-efficient solution for the pipelined ADC presented.

### 3.1. Stage-by-Stage Optimization of Resolution and Capacitance

In pipeline ADCs, the resolution of each stage significantly impacts the overall performance of the converter, influencing factors such as noise, linearity, and power consumption. From a noise perspective, let the equivalent input noise of the front-end sampling and holding circuit be denoted as $\bar{V_{ni,SH}^{2}}$, with a gain of $G_{SH}$. Similarly, let the equivalent noise of the i-th stage in the pipeline ADC be represented as $\bar{V_{ni,i}^{2}}$, with a gain of $G_{i}$. The total equivalent input noise of the pipeline ADC can then be expressed by the following Equation (1).

(1) $$\bar{V_{ni,tot}^{2}}=\bar{V_{ni,SH}^{2}}+\frac{\bar{V_{ni,1}^{2}}}{G_{SH}^{2}}+\frac{\bar{V_{ni,2}^{2}}}{{\left(G_{SH}G_{1}\right)}^{2}}+\ldots +\frac{\bar{V_{ni,M}^{2}}}{\prod_{i=1}^{M-1}G_{SH}^{2}G_{i}^{2}}.$$

Equation (1) shows that the noise contributed by each stage affects the total equivalent input noise of the ADC. Notably, the noise from the SHA circuit is directly coupled to the input of the ADC and can account for more than half of the total noise. Additionally, since $G_{SH}$ is typically set to 1, the noise contribution of the first stage has a substantial impact on the overall equivalent noise. However, the noise generated by subsequent stages, after being divided by the square of the product of the gains of all preceding stages, has a significantly diminished effect on the total equivalent noise of the entire system.

Next, from a linearity perspective, the relationship between the ADC’s differential non-linearity (DNL) and the capacitor mismatch error in the first stage is described by Equation (2):

(2) $$DNL=\frac{\Delta C_{i}\cdot 2^{N}}{C_{total}},$$

where $\Delta C_{i}$ denotes the error of each capacitor relative to its ideal value, $C_{total}$ represents the total capacitance of the first stage, and *N* is the ADC resolution. Each capacitor can be equivalently expressed as shown in Equation (3):

(3) $$C=\frac{C_{total}}{2^{m}},$$

where *m* represents the resolution of the first pipeline stage, and $C_{total}$ is constrained by the signal-to-noise ratio (SNR) of the ADC. In other words, for a fixed $C_{total}$, each one-bit increase in the resolution of the first pipeline stage results in a halving of the unit capacitance *C*. Assuming that the capacitance mismatch arises from random variations in the oxide thickness of the dielectric layer, denoted as *k*, we can derive Equation (4):

(4) $$\Delta C_{i}=k\sqrt{C}=k2^{-m/2}\sqrt{C_{total}},$$

Finally, by substituting Equation (4) into Equation (2), we obtain the expression in Equation (5).

(5) $$DNL=\frac{k\cdot 2^{(N-m)/2}}{\sqrt{C_{total}}}.$$

From Equation (5), it is evident that increasing the resolution *m* of the first stage by one bit or doubling the total sampling capacitance $C_{total}$ of the first stage results in a reduction in the DNL by a factor of $\sqrt{2}$.

Lastly, in terms of power consumption, reducing the capacitance in each stage lowers the charging and discharging power required by the sampling capacitors. This also alleviates the load on the operational amplifiers in each MDAC stage, which significantly contributes to the overall reduction in power consumption.

In pipelined ADCs, three common architectures are typically used: the 1.5-bit/stage MDAC, the 2.5-bit/stage MDAC, and the 3.5-bit/stage MDAC. First, if a 3.5-bit/stage MDAC was employed in the first stage, as analyzed above, it would effectively reduce the noise level. However, this architecture is highly complex, with a closed-loop feedback factor of approximately $\frac{1}{8}$, making it difficult to design an operational amplifier that meets the required precision specifications. As a result, the 3.5-bit/stage MDAC structure was not considered for this design. The 2.5-bit/stage MDAC, which requires six comparators, is more complex than the 1.5-bit/stage MDAC, which only requires two comparators. The decision levels for these comparators are inherently defined within the system, eliminating the need for additional circuitry. Therefore, adopting a 1.5-bit/stage pipelined structure for the first stage simplifies the front-end design, reducing both the chip area and power consumption.

Considering the overall noise tolerance of the pipeline ADC and balancing trade-offs between speed, power consumption, and area, we selected a 1.5-bit/stage MDAC structure for all stages. The advantages of this approach are as follows: First, it simplifies both the circuit and layout design, which helps shorten the design cycle. Second, as demonstrated in Equation (1), the output signal from each stage is further amplified by subsequent stages, thereby attenuating the noise contribution of later stages. This allows for a reduction in the capacitor size for the later stages. Lower capacitance is beneficial for optimizing overall power consumption. After careful consideration of capacitor matching principles, we propose the following solution: the internal capacitances of MDACs 1 to 4 remain unchanged to minimize the impact of noise; the capacitance values for MDACs 5 and 6 are reduced by a factor of 0.7; and the capacitance sizes for MDACs 7 and 8 are kept the same as those in the preceding stages, which also simplifies circuit design and layout. Third, with the uniform use of a 1.5-bit/stage MDAC structure across all stages, the calibration algorithm can be optimized for this fixed resolution, simplifying the digital correction process. This reduces the complexity of the digital correction logic, leading to lower area and power consumption, while simultaneously improving the system’s overall reliability. The final architecture is shown in Figure 2.

### 3.2. Removing Front-End Sample-and-Hold Amplifier (SHA-Less)

In a traditional pipeline ADC architecture, the front-end stage typically includes the sample-and-hold amplifier (SHA) module, which captures the full-scale input signal and is the most prone to introducing distortion. This stage generally consumes a significant amount of power, often accounting for more than 15% of the total ADC power consumption. Typically, the SHA operates with a gain of 1, meaning it does not suppress distortion or noise but may, in fact, introduce its own noise and harmonic distortions. However, for high-speed ADCs, the SHA is crucial, as it compensates for the timing mismatch between the two sampling networks—the first-stage MDAC and the sub-ADC—thereby reducing aperture errors in high-speed signals. The SHA thus minimizes signal errors resulting from these timing differences.

For the ADC proposed in this paper, however, which is designed for low-speed, low-power applications, the SHA is not essential. Given that the SHA does not directly contribute to the digital output and consumes considerable power, it can be omitted if the aperture error remains within an acceptable range. In this design, the first stage employs a 1.5-bit/stage MDAC architecture, which offers a wide digital error correction range ($\pm \frac{1}{4}V_{\mathrm{ref}}$). This broad correction range helps to mitigate aperture error and comparator offset error, making it feasible to eliminate the SHA in the first stage of the pipeline ADC. To ensure that the aperture error remains within a correctable range, the same clock is used for both sampling networks, effectively eliminating clock skew. Additionally, gate-boosted switches are employed to achieve a uniform on-resistance independent of the input signal, which improves linearity. The layout utilizes a symmetrical routing approach, and multiple extractions of parasitic parameters, followed by post-simulation, are performed to minimize clock sampling errors in both sampling paths under various process corners, power supply voltage fluctuations, and temperature variations. These considerations help maximize the reduction in aperture error effects.

### 3.3. Op-Amp Sharing Technique

In pipeline ADCs, the MDAC circuit plays a crucial role in overall performance. Typically, the op-amp is the most critical component within the MDAC circuit and also consumes the most power. Current low-power design strategies for pipeline structures primarily focus on reducing op-amp power consumption. Since the even and odd stages of the MDAC operate in different clock cycles, this design adopts op-amp sharing technology to minimize power consumption. By allowing adjacent stages of the MDAC circuit to share a single op-amp, power consumption is reduced by nearly 50%. However, traditional op-amp sharing structures in stage-shared MDACs introduce several key issues that impact system accuracy. First, in conventional op-amp sharing structures, the sharing switch, typically composed of NMOS transistors, suffers from charge injection and clock feedthrough effects due to parasitic capacitances. Additionally, the sharing switch introduces parasitic resistance, which, when combined with the op-amp’s input capacitance, forms a low-pass filter that negatively affects the settling behavior of the MDAC circuit. Second, op-amp sharing causes the op-amp to remain in either a holding or amplifying state, without resetting its input terminal. Due to the op-amp’s finite DC gain, offset voltage, and noise, the input parasitic capacitance retains residual values from the previous clock cycle, which accumulate over time, leading to errors that degrade circuit performance.

To address these issues, this paper proposes a novel op-amp-shared MDAC structure that utilizes a switched op-amp with dual input differential pairs to enable sharing between two stages. This design integrates the traditional external sharing switch into the op-amp itself. The sharing state is alternated by switching the internal sharing switch, which connects the shared op-amp between the previous and subsequent stages. The process works as follows: One pair of input differential pairs is used for amplification to produce an output, while the other pair samples the input value from the previous stage. These two pairs operate under the control of two overlapping clocks, which are in opposite phases. Since both pairs of input differential pairs are reset to the input common-mode voltage in each clock cycle, the accumulated errors typically seen in traditional op-amp sharing structures—due to the lack of a reset operation—are effectively eliminated.

### 3.4. Reducing Comparator Power

In the sub-ADC, the comparators are responsible for comparing input signals at each stage, consuming static current, and thereby increasing overall system power consumption. As the resolution of the pipeline ADC increases, the number of comparators increases exponentially. For the 10-bit ADC discussed in this paper, we implement a 1.5-bit-per-stage structure for the first eight stages, each using two comparators, resulting in a total of 16 comparators for these stages. The final stage is a two-bit flash ADC, which requires three comparators to resolve four quantization levels. Thus, the system comprises a total of 19 comparators. Optimizing the comparator design is crucial for reducing ADC power consumption and can lead to a significant reduction in overall system power. In our design, digital error correction is employed, allowing the maximum permissible offset error of the comparator to reach $\frac{1}{4}V_{\mathrm{ref}}$. To further optimize comparator power consumption, we chose a dynamic comparator design. Dynamic comparators consume negligible static power when there is no input signal and respond quickly during comparison, thereby significantly reducing average power consumption.

Given the absence of a SHA in this design and the need to maximize the permissible range of aperture error, we made several modifications to the dynamic comparators. In the first stage of the pipeline ADC, the sub-ADC utilizes a dynamic comparator with a pre-amplifier. This adjustment enhances input linearity, allowing the comparator to handle large signal swings more effectively while minimizing disturbances to the input signal. In subsequent stages, the signal output from the operational amplifier of the previous stage is sampled, ensuring high comparison accuracy and signal integrity.

From the second stage onward, a current-summing-based dynamic differential comparator is employed to further reduce the area and power consumption of the sub-ADC. This comparator structure balances power consumption and performance by summing the differential signal currents during the comparison process, thus reducing current fluctuations and optimizing energy efficiency. The comparator is clock-driven, activating only during necessary clock phases, which minimizes static power consumption during idle periods. This clock-driven operation is essential for reducing overall power consumption.

Additionally, a regenerative latch mechanism is integrated within the comparator to accelerate the output response, enabling rapid signal comparison and reset. This is crucial for maintaining high throughput in pipeline ADC applications, where speed is a key requirement. The current-summing design also incorporates an enhanced current mirror technique, ensuring that currents stabilize quickly to a balanced state. This guarantees both low power consumption and robust performance, even under high-frequency operation. Consequently, this design optimizes both the power and area of each sub-ADC stage while ensuring high comparison accuracy and fast response throughout the entire pipeline conversion process.

## 4. Circuit Implementation

### 4.1. MDAC with Shared Op-Amp

**Figure 3:**
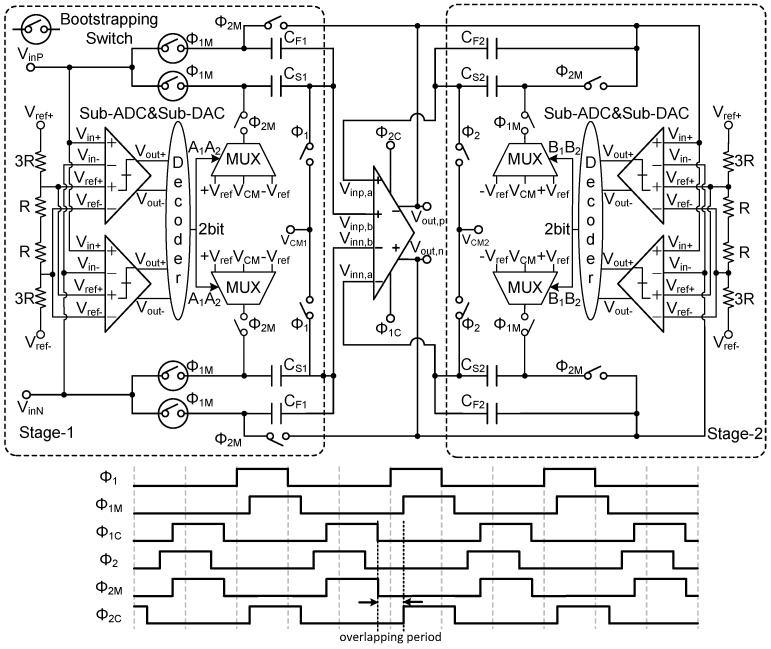
Structure and timing of first stage and second stage.

This paper presents a novel shared op-amp MDAC architecture, as illustrated in Figure 3. The architecture comprises several key components, including sampling, comparator quantization, digital-to-analog conversion, and gain amplification. At the core of this design is a dual-input differential pair with a switched op-amp. The functions of comparator quantization and digital-to-analog conversion are performed by a sub-ADC, which consists of two comparators with latch functionality and a digital decoder unit. Two reference voltages, $+\frac{1}{4}V_{\mathrm{ref}}$ and $-\frac{1}{4}V_{\mathrm{ref}}$, are compared against $V_{\mathrm{in}+}$ and $V_{\mathrm{in}-}$ in the comparators. The comparison results are latched and then processed by the digital decoder to generate the output code.

The timing diagram of the MDAC is shown in Figure 3, and the overall operational sequence is as follows: When the clock $\Phi_{1}$ is high, $V_{\mathrm{inp},b}$ and $V_{\mathrm{inn},b}$ are connected to the common-mode input voltage $V_{\mathrm{CM}1}$ for resetting. Once $\Phi_{1}$ becomes low and $\Phi_{1M}$ becomes high, two pairs of capacitors, $C_{S1}$ and $C_{F1}$, are connected to $V_{\mathrm{inP}}$ and $V_{\mathrm{inN}}$, initiating the signal sampling process. At this point, with $\Phi_{1C}$ low and $\Phi_{2C}$ high, the input voltages $V_{\mathrm{inp},a}$ and $V_{\mathrm{inn},a}$ in the op-amp are connected, and the op-amp operates in the amplification state of stage 2. Simultaneously, $V_{\mathrm{inP}}$ and $V_{\mathrm{inN}}$ are fed into the input of the stage-1 sub-ADC, starting the quantization process.

When $\Phi_{1M}$ becomes low and $\Phi_{2}$ becomes high, the op-amp inputs $V_{\mathrm{inp},a}$ and $V_{\mathrm{inn},a}$ are reset by connecting them to the common-mode input voltage $V_{\mathrm{CM}2}$. Then, as $\Phi_{2}$ becomes low and $\Phi_{2M}$ becomes high, with $\Phi_{2C}$ low and $\Phi_{1C}$ high, $V_{\mathrm{inp},b}$ and $V_{\mathrm{inn},b}$ in the op-amp are connected, and the op-amp enters the amplification state of stage 1. The sampling capacitors $C_{S1}$ are then connected according to the sub-ADC output to the $+V_{\mathrm{ref}}$, $V_{\mathrm{CM}}$, and $-V_{\mathrm{ref}}$ terminals. Along with the op-amp, $C_{S1}$ and $C_{F1}$ perform the signal subtraction and amplification functions of stage 1, generating the residue signal. This residue signal is subsequently sampled by capacitors $C_{S2}$ and $C_{F2}$.

**Figure 4:**
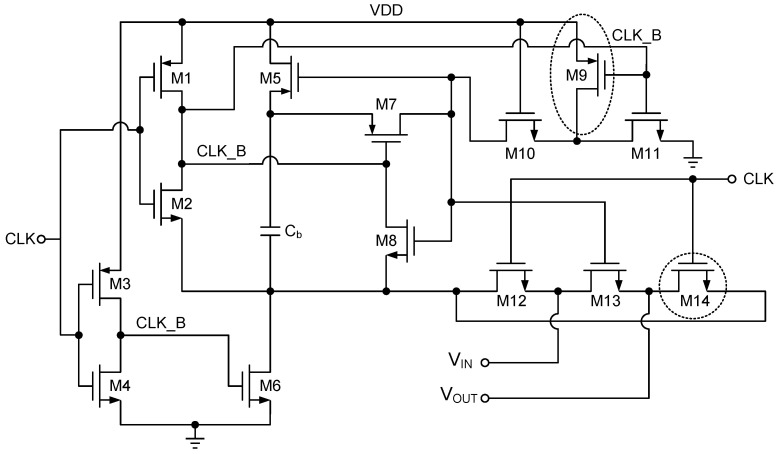
Structure of gate-boosted switch.

As shown in Figure 3, the MDAC circuit includes multiple switches, which are typically implemented using MOS transistors. The performance of these MOS switches has a significant impact on the overall circuit behavior, influenced by factors such as switching speed, resistance, charge injection, and clock feedthrough. In the first stage of the pipeline, both the MDAC and sub-ADC are directly connected to the continuous-time input signal. To enhance performance, all input sampling switches utilize gate-boosted technology. This approach effectively reduces the on-resistance of the switches and minimizes charge redistribution effects, ensuring that voltage variations in the signal path during switch operation are kept to a minimum. Consequently, this reduction in voltage fluctuations mitigates non-linear errors and improves conversion accuracy. Figure 4 illustrates the gate-boosted switch circuit used in this design.

The circuit is controlled by the clock signal, CLK. When CLK is low, CLK_B is high, and transistors M11 and M10 conduct, providing a discharge path for the main switch M13, thus turning M13 off. Meanwhile, the gate of M5 is pulled low by M10 and M11, causing M5 to conduct. Since M6 also conducts, the bootstrap capacitor, $C_{b}$, is charged to VDD. At this point, transistors M7 and M12 are turned off, isolating the bootstrap capacitor from the main switch, M13. When CLK transitions to high, CLK_B becomes low, M6 is turned off, and the gate of M7 is pulled low. The voltage difference between the gate and source of M7 becomes VDD, causing M7 to conduct. The charge stored in $C_{b}$ is then used to charge the gate of the switching transistor M13, enabling it to conduct. M12 and M14 are designed symmetrically to minimize charge asymmetry, ensuring a balanced operation. Additionally, M9 is employed to reduce non-linearity and parasitic capacitance at the drain node of M11, thereby improving the circuit’s stability.

### 4.2. Op-Amp

**Figure 5:**
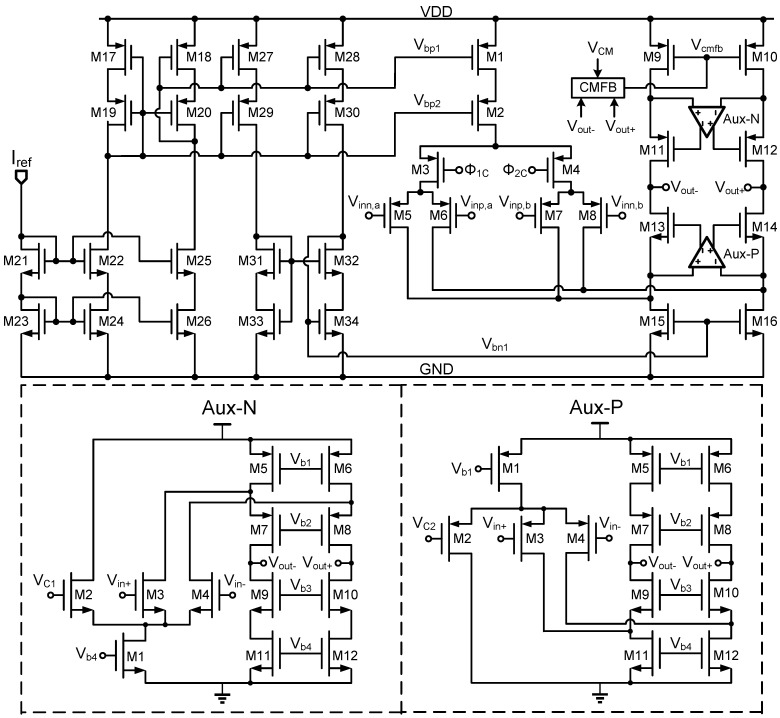
Dual-input, differential-pair, gain-boosted, switched operational amplifier circuit.

In the first-stage MDAC of the 1.5-bit/stage, 10-bit pipeline ADC, the op-amp must achieve a DC gain greater than 70 dB. To meet the performance specifications of the pipeline ADC presented in this paper while providing a large output swing, a gain-boosted folded-cascode amplifier is employed. The gain is enhanced using a gain-boosting circuit, and the two auxiliary op-amps are also based on the folded-cascode structure. The op-amp circuit is shown in Figure 5. The input stage consists of two sets of PMOS differential pairs, M5 and M6 and M7 and M8, which help to minimize noise. These pairs are connected to the input signals $V_{\mathrm{inn},a}$, $V_{\mathrm{inp},a}$, $V_{\mathrm{inp},b}$, and $V_{\mathrm{inn},b}$, respectively. The control signals $\Phi_{1C}$ and $\Phi_{2C}$ serve as switches to toggle the operating state of the two input pairs. When $\Phi_{1C}$ is low, switch M3 conducts, enabling M5 and M6 to act as the input transistors of the op-amp. Similarly, when $\Phi_{2C}$ is low, switch M4 conducts, allowing M7 and M8 to function as the input transistors.

**Figure 6:**
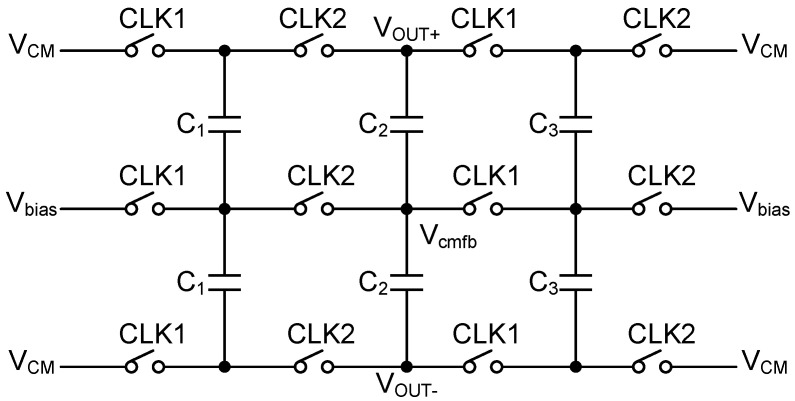
Switched-capacitor common-mode feedback circuit.

The fully differential architecture effectively minimizes imbalances caused by non-ideal factors. However, in feedback circuits, the introduction of differential signals can lead to uneven changes in the two branches, potentially causing the MOS transistors to move out of saturation and into the linear region. This transition can alter the amplifier’s gain, disrupting the proper operation of the circuit. To stabilize the common-mode voltage and mitigate this issue, a common-mode feedback (CMFB) circuit is employed. The CMFB circuit, shown in Figure 6, uses a simple switched-capacitor method. This approach is straightforward and consumes no DC power due to its dynamic operation. The CMFB circuit is controlled by two non-overlapping clocks, CLK1 and CLK2. During each clock cycle, capacitors alternately sample the common-mode voltage and transfer charge to the feedback node, ensuring that the common-mode voltage of the operational amplifier remains stable. Since the circuit does not have a continuous DC current path, it does not consume significant DC power. Power is only drawn during the dynamic charge–discharge cycle of the capacitors. Additionally, the MOS switches exhibit minimal leakage current when turned off, further contributing to the low power consumption. As a result, the CMFB mechanism ensures the stable operation of the differential amplifier without introducing substantial power loss, making it particularly well suited for low-power applications.

### 4.3. Comparator

**Figure 7:**
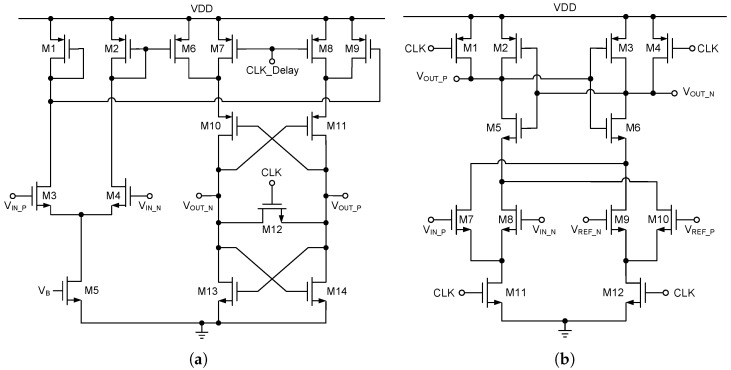
(**a**) The dynamic comparator with a pre-amplifier and (**b**) differential-pair dynamic comparator.

To maximize the allowable aperture error range inherent in the SHA-less architecture, a dual-stage comparator is utilized in the design of the first-stage sub-ADC, as illustrated in Figure 7a. This structure consists of a pre-amplifier in the first stage and a dynamic comparator in the second stage. During the pre-amplification phase, transistors M1–M5 function as the input stage, with M3 and M4 forming a differential amplifier. M5 serves as a current source, providing a constant current, while M1 and M2 act as active loads for the differential pair, enabling the amplification of the voltage difference between $V_{\mathrm{IN}\_P}$ and $V_{\mathrm{IN}\_N}$. In the latching phase, the cross-coupled pairs M10 and M11 and M13 and M14 form a positive feedback latch structure. This latch further amplifies the small voltage difference from the pre-amplification stage and rapidly converts it into full logic-level outputs, $V_{\mathrm{OUT}\_P}$ and $V_{\mathrm{OUT}\_N}$, while maintaining the output state once latched. M12 functions as the latch enable switch. When the clock signal CLK is active, M12 is turned on, causing the latch to enter its operational state and thereby latching the current comparison result. The CLK_Delay signal introduces a delay after the CLK signal, ensuring sufficient time for the latch to respond to the differential voltage from the input pair. This delay minimizes the risk of errors or instability during the latching phase due to excessive speed, ultimately improving the accuracy and stability of the comparator.

To further reduce power consumption in the sub-ADC, a dynamic comparator based on current summation principles is adopted from the second stage onward, as shown in Figure 7b. In the pre-charging phase, when the clock signal CLK is low, M1 and M4 conduct, and the output nodes are pre-charged to VDD. When CLK transitions to high, the circuit enters the comparison phase. The input differential voltage is converted into a voltage difference by two differential pairs, M7–M8 and M9–M10, along with their corresponding loads, which produce the comparison result. The threshold voltage of this comparator is determined by the W/L ratio of transistors M7–M10.

## 5. Measured Results

**Figure 8:**
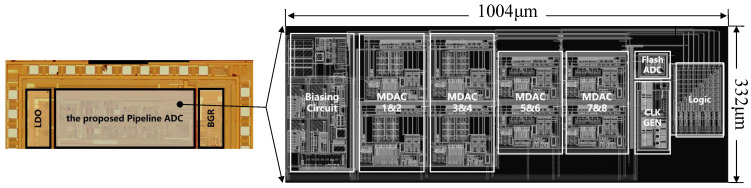
Die photo micrograph.

To verify the proposed circuit, a radio frequency (RF) chip for the IoT was fabricated using the TSMC 1P5M 180 nm CMOS process with MIM capacitor technology. The chip’s micrograph is shown in Figure 8, which features a common-centroid design to achieve the precise matching of sample and feedback capacitors, ensuring minimal errors due to mismatch in the capacitors. The pipeline ADC occupied 0.333 mm^2^ (1.004 mm × 0.332 mm). It consumed 5 mW of power at a 20 MS/s sampling rate from a 1.8 V supply, which included the energy used by the clock and reference circuits. This low power consumption, combined with the small area, makes it a promising candidate for low-power IoT applications.

**Figure 9:**
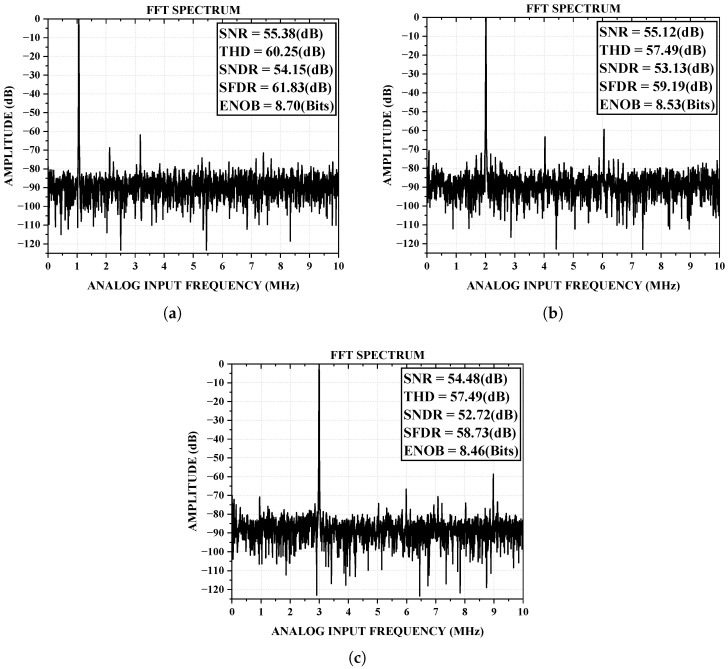
Measured ADC output FFT spectra under (**a**) $F_{S}$ = 20 MHz and $F_{IN}$ = 1MHz; (**b**) $F_{S}$ = 20 MHz and $F_{IN}$ = 2.4 MHz; and (**c**) $F_{S}$ = 20 MHz and $F_{IN}$ = 3.6 MHz.

Figure 9a presents the measured output spectrum at an input frequency of 1 MHz. The measured signal-to-noise and distortion ratio (SNDR) and spurious-free dynamic range (SFDR) were 54.15 dB and 61.83 dB, respectively. The total harmonic distortion (THD) at this frequency was 60.25 dB. These results indicate that the ADC was able to maintain high performance even at relatively low frequencies. However, it is important to note that while the THD was reasonably low, further optimization of the design could help to improve this parameter, especially in high-dynamic-range applications.

Figure 9b shows the spectrum of the ADC sampling a 2.4 MHz input, which achieved an SNDR of 53.13 dB and an SFDR of 59.19 dB. Similarly, when a 3.6 MHz input signal was applied, the ADC achieved an SNDR of 52.72 dB and SFDR of 58.73 dB, as shown in Figure 9c. These results demonstrate that the performance of the ADC remained stable across a range of input frequencies, though a slight degradation in the SNDR and SFDR could be observed at higher frequencies. This behavior was likely due to the limitations of the process technology and the inherent design trade-offs in the ADC architecture, such as the finite bandwidth of the op-amps and the sampling circuit.

**Figure 10:**
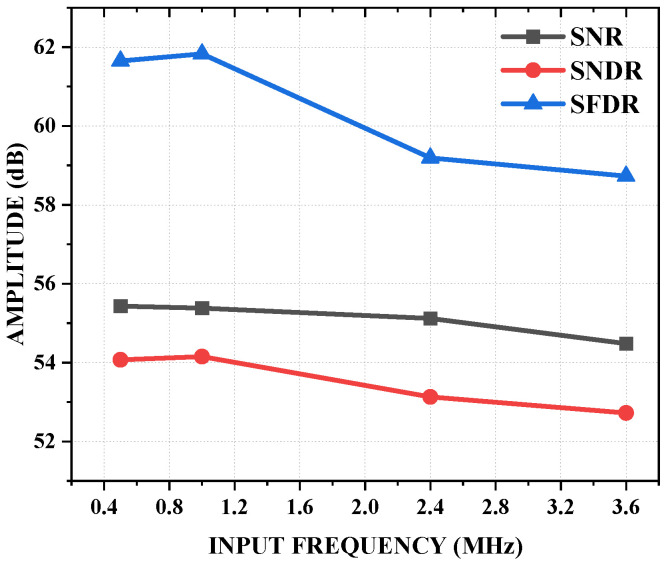
Measured SNR, SNDR, and SFDR versus input frequency at 20 MS/s.

**Figure 11:**
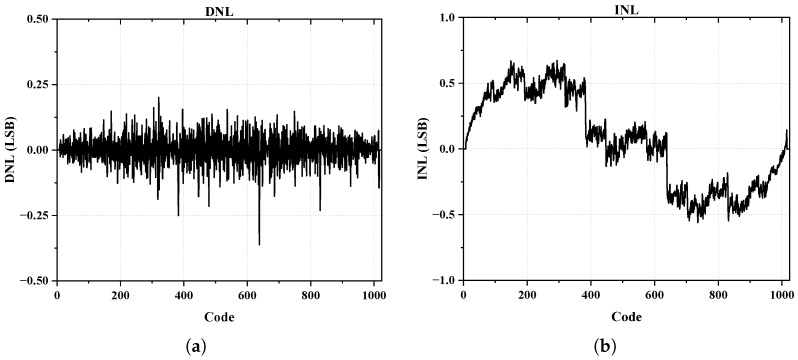
(**a**) The measured DNL of the proposed ADC and (**b**) measured INL of the proposed ADC.

Figure 10 presents the SNR, SNDR, and SFDR of the ADC across an input frequency sweep at a 20 MS/s sampling rate. The decrease in the SNDR and SFDR at higher frequencies was consistent with the results shown in Figure 9a–c, and it highlights the trade-off between performance and sampling speed. The static performance of the ADC was characterized by a measured maximum differential non-linearity (DNL) of 0.36 LSB and a maximum integral non-linearity (INL) of 0.67 LSB. Figure 11a,b display the measured DNL and INL, respectively. These non-linearity values indicate that the ADC achieved good linearity performance, which is critical for accurate signal conversion.

The performance of the ADC is compared to prior ADCs in Table 1. Compared to other ADCs, the prototype ADC achieved superior power efficiency and a smaller area, which is a significant advantage for IoT devices that require long battery life and low-cost systems. The smaller area also contributes to the overall reduction in system cost, making it an attractive solution for IoT applications.

It is worth noting that if advanced nanometer technology, such as 90 nm or 65 nm technology, was used to implement the ADC, better figures of merit (FOMs) and power efficiency could potentially be achieved. This is because advanced process nodes typically allow for higher transistor density, faster switching speeds, and lower power consumption, all of which could enhance the performance of the ADC. However, the current 180 nm process used in this work offers a good balance between performance and manufacturing cost, making it suitable for many IoT applications.

## 6. Conclusions

This paper proposes a 10-bit, 20 MS/s pipelined ADC implemented in a 180 nm CMOS, occupying a compact area of 0.333 mm^2^, which is notably smaller than typical designs fabricated in more advanced process nodes. By employing an SHA-less architecture, op-amp sharing, and dynamic comparators, this ADC achieves a low power consumption of only 5 mW, with a measured performance of an SFDR of 61.83 dB and SNDR of 54.15 dB.

A key enabler of this power efficiency and performance is a novel gain-boosted op-amp featuring a dual-input differential pair, which effectively mitigates crosstalk and memory effects inherent in SHA-less and op-amp sharing configurations. Combined with capacitor scaling and stage-wise optimization, this architecture offers an energy-efficient solution for IoT applications that demand low-to-medium-speed data conversion.

The compact size and demonstrated high performance make this ADC well suited for power-constrained IoT sensor nodes, particularly for applications such as vibration monitoring. Future work will focus on reducing power consumption through advanced circuit techniques, exploring higher-resolution versions, and investigating enhanced power management strategies to improve stability. Additionally, we plan to conduct performance evaluations in advanced CMOS nodes, such as the 90 nm process, to assess potential scaling benefits for future IoT systems.

In conclusion, this work presents a power-efficient, 10-bit, 20 MS/s pipelined ADC tailored for low-power IoT sensor nodes, offering a promising solution for battery-operated devices with extended lifespans.

## Figures and Tables

**Table 1:** Measured performance and comparison to prior work.

Parameter	This Work	[29]	[30]	[31]	[32]	[33]
Architecture	Pipeline	Pipeline	Pipeline	Pipelined SAR	Pipeline	Pipeline
Technology (nm)	180	130	500	180	130	110
Resolution (bits)	10	10	14	12	12	12
Area (mm^2^)	0.333	0.7	6.893	1.35	0.48	0.81
Sampling rate (MS/s)	20	200	30	10	40	30
Supply voltage (V)	1.8	1.2	5/3.3	1.8	3.3	3.3
SFDR (dB)	61.83	63	83.61	78.5	76.23	75.68
SNDR (dB)	54.15	53	75.51	65.8	/	71.17
INL (LSB)	0.67	1.05	1.82	1.67	0.6	/
DNL (LSB)	0.36	0.83	0.74	0.97	0.6	/
Power (mW)	5	38	136	12	60	44
FOM (pJ/conv-step)	0.59	0.552	0.931	0.75	0.517	/

## Data Availability

The data presented in this study are available on request from the corresponding author.
